# Erythropoietin production in renal cell carcinoma and renal cysts in autosomal dominant polycystic kidney disease in a chronic dialysis patient with polycythemia: A case report

**DOI:** 10.3892/ol.2014.2469

**Published:** 2014-08-21

**Authors:** KEIICHI ITO, TAKAKO ASANO, SUSUMU TOMINAGA, HIDEHIKO YOSHII, HARUTAKE SAWAZAKI, TOMOHIKO ASANO

**Affiliations:** 1 Department of Urology, National Defense Medical College, Tokorozawa, Saitama 359-8513, Japan; 2 Department of Pathology, National Defense Medical College, Tokorozawa, Saitama 359-8513, Japan

**Keywords:** autosomal dominant polycystic kidney disease, erythropoietin, renal cell carcinoma, immunohistochemistry, polycythemia

## Abstract

In patients undergoing chronic hemodialysis (HD), erythropoietin (EPO) production from the kidney generally decreases and renal anemia develops. Patients without anemia, but with high serum EPO (sEPO) levels are rare among HD patients. The current study presents the case of a 67-year-old female HD patient with autosomal dominant polycystic kidney disease (ADPKD) and renal cell carcinoma (RCC), manifesting polycythemia with elevated sEPO levels. A radical nephrectomy was performed, which diminished the polycythemia, but the sEPO levels remained high. To determine the origin of the EPO production, immunohistochemistry was performed to detect EPO in the RCC and the renal cysts of the surgically resected kidney. In addition, the sEPO and EPO levels in a renal cyst were determined by enzyme immunoassay. EPO expression was demonstrated in RCC and cyst epithelial cells using immunohistochemistry, revealing extremely high EPO levels in the cyst fluid. Due to the remission of polycythemia following the nephrectomy, EPO production from the resected kidney appeared to have been the cause of the polycythemia. Positive EPO staining of the renal cysts in the resected polycystic kidney and sustained sEPO elevation following nephrectomy led to the hypothesis of EPO production in the renal cysts of the contralateral polycystic kidney. Although the postoperative EPO level was higher than the normal range, the hematocrit (Hct) level gradually decreased and recombinant human EPO was required again three months following the nephrectomy. Eight months after the nephrectomy, the Hct level was 30.2% with the use of rHuEPO. In conclusion, EPO production from RCC and renal cysts in ADPKD appeared to cause polycythemia in the HD patient.

## Introduction

Polycythemia is one of the paraneoplastic syndromes associated with renal cell carcinoma (RCC), which has been associated with erythropoietin (EPO) production from renal carcinoma cells (1). Although the serum EPO (sEPO) level is reportedly elevated in 33–38% of patients with RCC, it is relatively rare that patients with RCC manifest polycythemia (2). The sEPO level is used as a tumor marker in patients with RCC, as it has been found to correlate with the stage and grade of RCC and provides prognostic information (2,3). A previous study has confirmed that the expression of EPO receptors and endogenous EPO by RCC cells stimulates RCC cell proliferation (4). In addition to RCC, several types of cancer cells reportedly use the EPO system for cell growth and angiogenesis (5).

sEPO levels are generally lower in patients undergoing chronic dialysis than in healthy individuals due to impaired EPO production by the renal cells. Five cases of EPO-producing RCC have previously been reported in patients undergoing chronic hemodialysis (HD) (6–9). These cases exhibited elevated hematocrit (Hct) and hemoglobin (Hgb) levels, but did not manifest polycythemia. Polycythemia in patients with RCC arising in end-stage kidney disease is a considerably rare event. Four of the five cases were associated with acquired cystic disease of the kidney (ACDK), and none of the cases were associated with autosomal dominant polycystic kidney disease (ADPKD) (6–9). RCC arising from ADPKD is an extremely rare condition (10).

We have previously reported the radiological finding of an ADPKD patient with RCC who manifested polycythemia (11). Although the polycythemia was diminished following the removal of the affected kidney, the sEPO levels remained elevated in the patient. This clinical course led us to consider EPO production in the contralateral polycystic kidney, as it was possible that not only RCC, but also renal cysts in ADPKD produce EPO. Therefore, in the current study, EPO production by RCC and renal cysts was analyzed in the surgically resected polycystic kidney, by immunohistochemistry and enzyme immunoassay (EIA). EPO production was observed in RCC and the renal cysts in ADPKD. This study also discussed the implications of sEPO levels at each time-point of the clinical course. As polycythemia diminished following nephrectomy, EPO production from the resected kidney appeared to cause polycythemia. Positive EPO staining of renal cysts in the resected polycystic kidney and sustained sEPO elevation following the nephrectomy allowed us to predict EPO production in the renal cysts of the contralateral polycystic kidney. Written informed consent was obtained from the patient.

## Case report

### Materials

Polyclonal goat anti-human EPO antibody was purchased from Santa Cruz Biotechnology, Inc. (Santa Cruz, CA, USA). The ChemMate ENVISION kit for immunohistochemical analysis was purchased from DakoCytomation (Kyoto, Japan).

### Patient clinical course

This study presents the case of a 67-year-old female undergoing chronic HD due to ADPKD manifesting polycythemia in 2003. As the patient’s sEPO level was elevated and abdominal computed tomography (CT) indicated an enhanced lesion that was 3 cm in diameter in the lower part of the left kidney, the patient was referred to the Department of Urology at the National Defense Medical College (Tokorozawa, Japan). The patient had a previous history of HD that began at the age of 58 years due to ADPKD. The patient presented with dilated cardiomyopathy and moderate grade mitral regurgitation, and thus, experienced severe heart dysfunction, including a decreased ejection fraction (25–30%) and diffuse hypokinesis of the cardiac walls. Prior to 2003, recombinant human EPO (rHuEPO) had been intermittently used to treat renal anemia. Although rHuEPO treatment was stopped due to the improvement of anemia in June 2003, the patient’s Hgb and Hct levels remained elevated. The Hct and Hgb levels reached 53.4% (normal range, 34.0–44.0%) and 16.5 g/dl (normal range, 12.0–15.0 g/dl), respectively, in September 2003 (Table I). The EPO level was 372 mU/ml (normal range, 8–36 mU/ml), and CT exhibited a considerably enhanced lesion in the left polycystic kidney. The radiological finding of this case has been previously reported (11).

**Figure 1:**
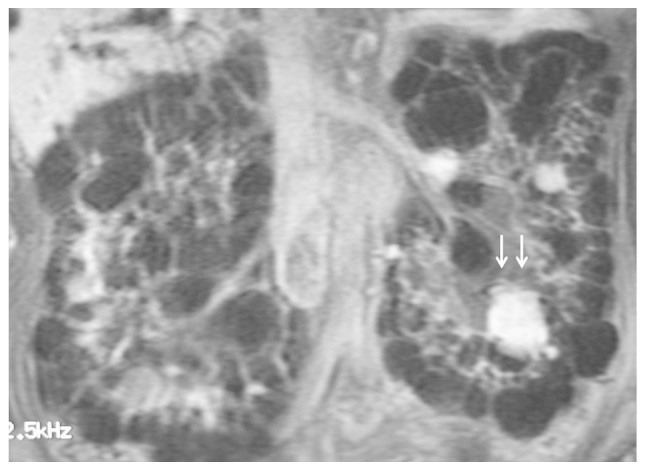
Magnetic resonance imaging (coronal view) revealing a highly enhanced tumor (white arrows) in the lower part of the left polycystic kidney.

**Figure 2:**
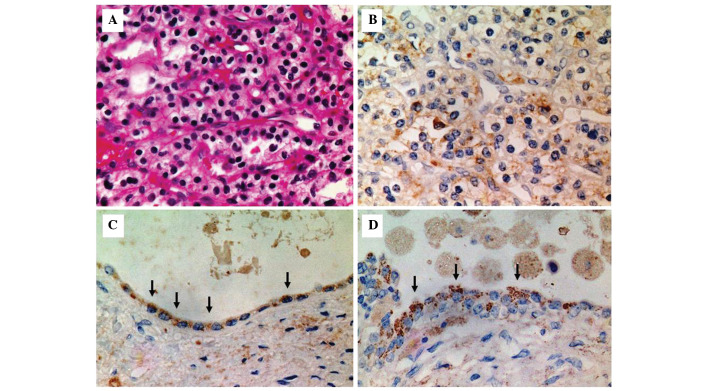
(A) Microscopic findings of the renal tumor with hematoxylin-eosin staining revealing a diagnosis of clear cell renal cell carcinoma (histological grades 1 and 2; pT1a). (B–D) Immunohistochemical analysis using anti-EPO antibody. (B) Positive staining was observed in the cytoplasm of the cancer cells. (C) Cells lining the cyst wall were positively stained with anti-EPO antibody (arrows), as well as those in the cytoplasm. (D) Positively-stained particles (arrows) were detected in the cytoplasm of the cyst-lining cells. EPO, erythropoietin.

At the time of admission, the patient exhibited erythema on the face and hands, possibly due to polycythemia. The laboratory results indicated high levels of Hgb (17.4 g/dl), Hct (57.1%), and an elevated EPO level (893 mU/ml) (Table I). CT, magnetic resonance imaging (Fig. 1) and Doppler ultrasonography indicated a hypervascular tumor (11). Although RCC was suspected, the possibility of a benign tumor, such as renal hemangioma, could not be excluded. Venous samples from bilateral renal veins were obtained, and as the EPO levels from the two renal veins were similarly elevated (right renal vein, 173 mU/ml; left, 159 mU/ml), the origin of EPO production could not be determined. For therapeutic and diagnostic purposes, an embolization and subsequent needle biopsy of the tumor were performed. Immediately after feeding, the artery was completely embolized by the superselective method (11) and a fine-needle biopsy was performed under ultrasonography. Four days after the embolization, the EPO level had decreased to 61.2 mU/ml. However, this level increased again to 1740 mU/ml eight days after the embolization (Table I). As the CT that was performed immediately after the marked increase in EPO level indicated a highly enhanced tumor, and the blood flow of the artery feeding the tumor appeared to have returned. With regard to the decrease in EPO level following the embolization, it was suggested that perhaps the renal lesion was producing EPO. Histological analysis of the biopsy specimen indicated that low-grade RCC was most likely. Although the patient had severe cardiac dysfunction, a left radical nephrectomy was performed to control the polycythemia. The surgical specimen exhibited a yellowish solid tumor in the lower part of the polycystic kidney. Microscopically, the tumor was diagnosed as RCC (clear-cell; grades 1 and 2; pT1a) (Fig. 2A). Post-operatively, the sEPO level decreased to 243 mU/ml (Table I), and although the post-operative EPO level remained higher than the normal range, the Hct level gradually decreased and rHuEPO was required again three months after the nephrectomy. Eight months after the nephrectomy, the Hct level was 30.2% with the administration of rHuEPO.

### Samples

Serum samples were obtained from the patient at each time-point of the clinical course and stored at −80°C until use. Cyst fluid was obtained immediately from the surgically resected specimen and stored at −80°C until use. A surgically resected tissue sample was fixed in 10% neutral-buffered formalin (Wako Pure Chemical Industries, Ltd., Osaka, Japan) for immunohistochemical analysis.

### Detection of EPO levels in serum and cyst fluid

A 50 ml aliquot of the serum samples and cyst fluid was used for the EPO immunoassay, which was performed in duplicate. EPO levels were quantified using an EIA kit (Diagnostic Products Corporation, Los Angeles, CA, USA) according to the manufacturer’s instructions.

### Immunohistochemical analysis in paraffin-embedded tissues

Immunohistochemical analyses to detect EPO were performed on RCC and renal cysts. A surgically resected left polycystic kidney was fixed in 10% neutral-buffered formalin and embedded in paraffin. Paraffin-embedded sections (4 μm) were mounted onto glass slides. The sections were dewaxed in xylene (Wako Pure Chemical Industries, Ltd.), rehydrated in decreasing concentrations of ethanol (Wako Pure Chemical Industries, Ltd.) and washed three times in phosphate-buffered saline (PBS) (Nissui Pharmaceutical Co., Ltd., Tokyo, Japan) for 10 min. Endogenous peroxidase was quenched for 45 min with 0.6% hydrogen peroxide in methanol (Wako Pure Chemical Industries, Ltd.). For antigen retrieval, subsequent to being washed with filtered water, the slides were boiled in 10 mmol/l citrate buffer (pH 6.0) for 20 min in an autoclave (121°C). Subsequent to being washed with PBS, a blocking step was performed using 3% bovine serum albumin (Wako Pure Chemical Industries, Ltd.) for 10 min. The primary goat polyclonal anti-human EPO antibody (Santa Cruz Biotechnology, Inc.) was subsequently incubated at 4°C overnight. The primary antibody was used at a dilution of 1:100. Following a second wash with PBS, the ChemMate Envision antibody (DakoCytomation) against goat immunoglobulins was used as a secondary antibody for 1 h. Sections were developed with diaminobenzidine and counterstained using 10% hematoxylin. Samples incubated without the primary antibody followed the same staining steps and were used for baseline staining.

### Results

#### Detection of EPO concentration in serum and cyst fluid

The sEPO levels determined by EIA are shown in Table I. The sEPO level significantly decreased following the embolization of the renal tumor, suggesting EPO production by the renal tumor. However, the sEPO level increased significantly several days later, suggesting that the blood flow to the tumor had been restarted. Although the EPO level decreased following the nephrectomy, the post-operative sEPO levels remained higher than the normal range. In addition, the renal cyst fluid exhibited an extremely high EPO concentration of 2,680 mU/ml in the surgically resected polycystic kidney, suggesting EPO production from the renal cysts.

#### Immunolocalization of EPO in renal cancer cells and cyst epithelial cells

EPO-positive staining was exhibited in the renal cancer cells (Fig. 2B) and the cells lining the cyst wall (Fig. 2C and D), as shown by immunohistochemical analysis using an anti-EPO antibody. Clear cell renal cancer and positive EPO staining were observed in the cytoplasm of the tumor cells (Fig. 2B). In the renal cysts of ADPKD, positive staining was located in the cytoplasm of the cyst epithelial cells (Fig. 2C and D) and positively stained particles were observed in the cytoplasm of the cells lining the cyst (Fig. 2D). Samples incubated without the primary antibody did not exhibit any staining (data not shown).

## Discussion

EPO production has been reported in several diseases, including hepatocellular carcinoma, hemangioblastoma of the cerebellum, gastric and pancreatic cancer, and in kidney lesions, such as RCC, renal cysts (6,9) and hemangioma (12). In the present case, no suspicious lesion was observed on imaging examination that could have produced EPO in other organs. However, the exact origin of the abnormal EPO production could not be determined by blood sampling from the bilateral renal veins. In addition, as the lesion in the left polycystic kidney was extremely hypervascular, there was also a possibility of a benign renal tumor, such as a hemangioma. EPO production from a renal hemangioma has been previously reported (12), therefore, a biopsy of the lesion was performed. Superselective embolization of the tumor significantly decreased the sEPO level, suggesting that the hypervascular lesion in the left kidney may be the origin of abnormal EPO production. As the lesion was suspected to be RCC and the likely cause of polycythemia, a surgical resection was indicated.

Following the superselective embolization, the decline of the EPO level with time, and its subsequent elevation, were observed. This may be due to EPO release by destroyed cells into the blood stream following embolization, or the induction of EPO production by hypoxia in renal cancer cells and/or other renal cells, such as cyst epithelial cells. Hypoxia inducible factor-1 (HIF-1) is reportedly upregulated in renal cancer cells and is important in cell proliferation and angiogenesis (13). HIF-1 transactivates genes encoding target proteins, including EPO, vascular endothelial growth factor and inducible nitric oxide synthase (14–16). The expression of HIF-1 and its target proteins, including EPO, may be increased by embolization. Considering the decline in EPO levels following the embolization and then the subsequent increase, the renal lesion was considered to be at least one of the causes of EPO elevation in the present study.

The EPO level remained above the normal range following the nephrectomy. As the production of EPO was observed in the cyst walls of the nephrectomized kidney and as bilateral elevation of sEPO level was demonstrated in the renal vein sampling, it is possible that the EPO may have been produced by cysts in the contralateral kidney. In addition, the EPO level following the left nephrectomy was higher than that four days after the embolization. Therefore, certain compensatory changes may have occurred in the contralateral polycystic kidney following the nephrectomy. Under normal conditions, compensatory changes following uninephrectomy of the contralateral kidney, including hypertrophy, are observed (17). Compensatory changes in the contralateral kidney of patients with ADPKD have not been previously evaluated, thus, the phenomenon of the sustained high EPO level following the nephrectomy is noteworthy. Furthermore, the reason for the decrease in the post-operative Hct level, in spite of an EPO level higher than the normal range, is unknown. An extremely large amount of EPO may be required to manifest polycythemia.

In normal kidneys, EPO is produced in the peritubular cells under the control of an oxygen sensor located in the epithelial cells of the proximal tubules (18). In previous studies, EPO production has been observed in RCC cells and epithelial cells in the cyst wall of patients with RCC arising from ACDK (6,9). In the present case, EPO production was also demonstrated by immunohistochemical analysis in the RCC and cyst epithelial cells. The high EPO levels in the cyst fluid further confirmed EPO production in the renal cysts. EPO production by RCC cells and cyst epithelial cells in the left kidney may cause polycythemia. Patients with ADPKD undergoing chronic dialysis have exhibited significantly higher EPO levels compared with patients without ADPKD (19). Furthermore, ACDK has also been associated with the improvement of anemia in patients on long-term HD (20). Therefore, renal cysts occasionally produce EPO in patients undergoing chronic dialysis. In addition, a high risk of RCC development has been reported for ACDK patients (21,22), indicating that EPO production in renal cysts may be involved in RCC development.

In conclusion, the present study demonstrated EPO production in RCC arising from ADPKD and the renal cysts of ADPKD. The extremely high EPO concentration in the cyst fluid supported the hypothesis of EPO production from the renal cysts in ADPKD. Based on the results of immunohistochemistry, EIA and the clinical course, polycythemia was caused by high EPO production from the resected kidney. The sustained elevation of sEPO may have been due to EPO production from the renal cysts in the contralateral polycystic kidney.

## Figures and Tables

**Table I:** Levels of sEPO, Hct and Hgb at various time-points.

Blood tests	Mar 2003	Sept 2003	Oct 2003 (Admission)	Nov 17, 2003 (4 days post-EMB)	Nov 21, 2003 (8 days post-EMB)	Dec 16, 2003 (post-RNx)	Jan 2004 (1 month post-RNx)	Mar 2004 (3 months post-RNx)
sEPO, mU/ml	NE	372	893	61.2	1740	243	241	NE
Hct, %	29.2	53.4	57.1	45.9	NE	35.0	35.0	31.7
Hgb, g/dl	8.3	16.5	17.4	14.3	NE	10.6	10.3	9.2

EMB; embolization, RNx; radical nephrectomy, sEPO, serum erythropoietin; Hct, hematocrit; Hgb, hemoglobin; NE; not examined.
